# The viral distribution and pathological characteristics of BALB/c mice infected with highly pathogenic Influenza H7N9 virus

**DOI:** 10.1186/s12985-021-01709-7

**Published:** 2021-11-29

**Authors:** Xiao-Xin Wu, Song-Jia Tang, Shu-Hao Yao, Yu-Qin Zhou, Lan-Lan Xiao, Lin-Fang Cheng, Fu-Ming Liu, Nan-Ping Wu, Hang-Ping Yao, Lan-Juan Li

**Affiliations:** 1 State Key Laboratory for Diagnosis and Treatment of Infectious Diseases, National Clinical Research Centre for Infectious Diseases, Collaborative Innovation Center for Diagnosis and Treatment of Infectious Diseases, The First Affiliated Hospital, Zhejiang University School of Medicine, 79 Qing Chun Road, Hangzhou, 310003 Zhejiang China; 2 Plastic and Aesthetic Surgery Department, Affiliated Hangzhou First People’s Hospital, Zhejiang University School of Medicine, Hangzhou, 310000 Zhejiang China; 3 Department of Stomatology, Wenzhou Medical University Renji College, Wenzhou, 325035 Zhejiang China; 4 Department of Respiratory Medicine, The Sir Run Run Shaw Hospital, Zhejiang University School of Medicine, Hangzhou, 310016 Zhejiang China

**Keywords:** Highly pathogenic H7N9, Multiple organ injury, Viral distribution, Pathological changes

## Abstract

**Background:**

The highly pathogenic Influenza H7N9 virus is believed to cause multiple organ infections. However, there have been few systematic animal experiments demonstrating the virus distribution after H7N9 virus infection. The present study was carried out to investigate the viral distribution and pathological changes in the main organs of mice after experimental infection with highly pathogenic H7N9 virus.

**Methods:**

Infection of mice with A/Guangdong/GZ8H002/2017(H7N9) virus was achieved via nasal inoculation. Mice were killed at 2, 3, and 7 days post infection. The other mice were used to observe their illness status and weight changes. Reverse transcription polymerase chain reaction and viral isolation were used to analyse the characteristics of viral invasion. The pathological changes of the main organs were observed using haematoxylin and eosin staining and immunohistochemistry.

**Results:**

The weight of H7N9 virus-infected mice increased slightly in the first two days. However, the weight of the mice decreased sharply in the following days, by up to 20%. All the mice had died by the 8th day post infection and showed multiple organ injury. The emergence of viremia in mice was synchronous with lung infection. On the third day post infection, except in the brain, the virus could be isolated from all organs (lung, heart, kidney, liver, and spleen). On the seventh day post infection, the virus could be detected in all six organs. Brain infection was detected in all mice, and the viral titre in the heart, kidney, and spleen infection was high.

**Conclusion:**

Acute diffuse lung injury was the initial pathogenesis in highly pathogenic H7N9 virus infection. In addition to lung infection and viremia, the highly pathogenic H7N9 virus could cause multiple organ infection and injury.

## Background

Virus infection is a serious human health problem, and influenza virus is one of the major pathogenic viruses [1, 2]. Over the past 100 years, there have been many influenza epidemics, with the Spanish flu outbreak in the early twentieth century being the worst. In the past 20 years, there have also been several serious influenza infection outbreaks. In 1997, high pathogenic H5N1 avian influenza virus crossed the species barrier to infect humans [3]. To date, the H5N1 outbreak has caused more than 800 infections, with more than 400 deaths, with a mortality rate of more than 50%. In 2009, the H1N1 pandemic appeared in the American continent. Since 2009, the H1N1 pandemic has spread worldwide, killing more than 200,000 people. In 2013, the novel H7N9 avian virus started to infect humans, causing more than 1500 infections, with a fatality rate as high as 40% [4]. In 2017, the H7N9 virus mutated and changed to a highly pathogenic H7N9 virus [5, 6]. Since then, 32 cases of human infection with highly pathogenic H7N9 virus have occurred in eight provinces in China, with a mortality rate of around 50% [7]. Fortunately, in order to control and eradicate the H7N9 virus, many actions had been taken including closing live poultry markets and culling poultry, standard bioinformatics analysis for monitoring virus evolution, the establishment of the platform for influenza research, early-warning, and introduction of the bivalent H5/H7 vaccine in poultry [8–13]. Several studies reported that the use of the poultry vaccine could reduce the H7N9 virus prevalence in poultry and successfully eliminate the human infection with H7N9 virus [11–13]. However, the H7N9 virus has not been eradicated in poultry in China, and might escape from the vaccine-induced immunity [14]. At the same time, the influenza virus is still changing, different viruses are emerging, and a novel H7N9 virus might appear again, causing new threats to human health [14].

Many clinical and epidemiological studies have shown that H7N9 displays obvious family aggregation, indicating that close contact between people might lead to H7N9 infection [15–19]. Once H7N9 increases its adaptability to human receptors to acquire human transmission ability, it will lead to an influenza epidemic and mass infection [20]. Fortunately, a recent study reported that the highly pathogenic H7N9 viruses isolated from poultry between 2018 and 2019 have weakened ability to bind to human-type receptors but high affinity to bind to avian-type receptors [14]. Highly pathogenic H7N9 viruses isolated from avian species have low-to-moderate pathogenicity in mice, but after replication in mammalian hosts, the H7N9 influenza viruses could easily acquire the mutations (PB2 627 K or PB2 701 N) and then become more virulent in mice [5, 13, 14]. With the evolution and recombination, the highly pathogenic H7N9 viruses may also has the potential to infect human. Mortality could also increase because of mutations in highly pathogenic H7N9 virus. Therefore, on the one hand, we should study the affinity of the virus to receptor, and on the other hand, we should also study the pathogenic characteristics of the virus, especially its multiple organ distribution and pathological characteristics after virus infection.

Many influenza viruses have been reported have acquired an extra-pulmonary infection ability [21, 22]. Clinically, the most frequently described symptoms are viral encephalitis and viral myocarditis [1, 23, 24]. The H1N1 virus has been confirmed to cause viremia, which led to brain infection. H5N1 has also been confirmed to cause viremia [25]. Viremia could more often be detected in fatal cases rather than in nonfatal cases [26]. H5N1 pathogenesis is characterised by intense inflammatory responses and a high viral load. The morbidity of H5N1 viruses is associated with their extra-pulmonary [27–30]. The plasma of patients was shown to contain H7N9 virus RNA; however, this did not correlate with the clinical outcome. H7N9 virus RNA could also be detected in urine and faeces [31]. We have confirmed the presence of viremia in highly pathogenic H7N9 infections. The initial infection site of highly pathogenic H7N9 is the lung. Lung infection is the main manifestation, and it will eventually develop to multiple organ failure [32]. Patients infected with H7N9 virus developed cytokine storms and viremia, and died from multiple organ failure. Whether multiple organ failure is an inflammatory response caused by lung infection, or a later viral, multi-organ infection is unclear, and solving this problem would guide treatment. There is currently limited clinical and pathological research on H7N9 virus infection, and there have been few systematic animal experiments to prove the virus distribution after highly pathogenic H7N9 virus infection [33]. To answer the above questions, we designed experiments in mice infected with highly pathogenic H7N9 viruses to clarify the viral distribution and pathological changes to important organs after viral infection.

## Methods

### Animal experiment ethical statement

Specific-pathogen-free (SPF) female BALB/c mice (n = 31, 6–8 weeks old) were obtained from the Experimental Animal Center of Zhejiang Province, China. All animal experiments were carried out following the principles of the Guide for the Care and Use of Laboratory Animals of Zhejiang Province. The Ethics Committee of the First Affiliated Hospital, Zhejiang University School of Medicine, approved the present study. A bio-safety level 3 laboratory at the First Affiliated Hospital, Zhejiang University School of Medicine was used to perform all the H7N9 virus experiments (Registration No. CNAS BL0022).

### Viruses and cells

The American type culture collection (Rockville, MD, USA) provided the Madin-Darby canine kidney cell line (MDCK). MDCK cells were cultured using Dulbecco's modified Eagle's medium (DMEM; Cat#11965092, Gibco, Grand Island, NY, USA) containing 10% foetal bovine serum (FBS; Cat#10100147, Thermo Fisher Scientific, Waltham, MA, USA) at 37 °C in a 5% CO_2_ atmosphere. The present study used a A/Guangdong/GZ8H002/2017(H7N9) virus (GenBank: MF455313-455320) isolated from an infected patient in Guangzhou, China, in 2017. Viruses were grown in the allantoic cavities of 9-day-old SPF embryonated chicken eggs at for 72 h 37 °C. The harvested allantoic fluid was tested using a haemagglutinin (HA) assay. Fifty microliters of allantoic fluid was diluted at 1:2 using phosphate-buffered saline (PBS; Cat#20012500BT, Gibco) in a 96-well blood coagulation plate. Then, an equal volume of 1% chicken red blood cells were added, and observed at room temperature for 30–45 min. The highest dilution at which blood coagulation appeared was the HA titre. Allantoic fluid aliquots containing the virus were placed in a − 80 °C freezer until further use.

### Virus median tissue culture infectious dose (TCID_50_) determination

MDCK cells were inoculated into 96-well cell culture plates at 3 × 10^4^ cells/well (in 100 μl). The virus was diluted in viral growth liquid 10 times continuously, from 10^–1^ to 10^−10^, and the last two rows were reserved for the controls. After cells grew into a single layer in the 96-well plate, the medium was removed, the wells were rinsed with sterile PBS once, and the diluted virus (100 μl/well) was added to the wells, with each concentration infecting cells in four wells. A normal cell control well (virus free) was set. The 96-well plate was incubated at 37 °C in a 5% CO_2_ incubator for 2 h, and then washed two times with PBS. The normal control well and the virus infection wells then received virus growth fluid (DMEM with 1% FBS; 100 μl/well). The microtitre plate was incubated for 72 h in a 5% CO_2_ incubator at 37 °C. The HA assay was then used to identify positive or negative wells. The TCID_50_ was calculated according to a previously published method [34].

### Inoculation of the virus

Fifty microliters of 10^6^ TCID_50_ of A/Guangdong/GZ8H002/2017(H7N9) virus was inoculated into the mice intranasally. The control group (n = 5) received the same volume of PBS. The mice (n = 14) were observed for signs of death, weight loss, and illness post-infection. Some mice (n = 12) were sacrificed humanly at 2, 3, and 7 days post infection (dpi). Their serum and organs (spleens, livers, kidneys, hearts, brains, and lungs) were excised or collected. The organs were dived into parts, one of which was fixed in 10% buffered formalin, and the other subjected to viral isolation and quantitative PCR to detect virus levels.

### Organ histopathology

Haematoxylin eosin (HE) staining of organ tissues was performed. The organ tissues were also subjected to Immunohistochemistry (IHC) staining. Organ tissues were embedded in paraffin and sectioned. The sections were then de-waxed and heated in citrate buffer. H_2_O_2_ (0.3%) in methanol was used to quench endogenous peroxidase activity. Sections were blocked for 2 h with Three percent bovine serum albumin (BSA; Cat#H1130, Solarbio, Tongzhou, Beijing, China) in PBS was used to block the sections for 2 h. The sections were then incubated at 4 °C overnight (12 h) with a 1:200 dilution of rabbit polyclonal antibodies recognizing H7N9 (Cat#GTX125989, GeneTex, Irvine, CA, USA). EnVision System reagents (Cat#K5007, DAKO, Glostrup, Denmark) were used to detect the bound antibodies. HE was used to counterstain all the slides.

### Virus isolation from mouse serum and organs

Virus isolation was attempted from serum sampled at 2, 3, and 7 dpi. The allantoic cavities of 9-day-old SPF embryonated chicken eggs were injected with approximately 100 µl of serum and cultured in an incubator for 72 h at 37 °C. Thereafter, the allantoic fluid was sampled and subjected to an HA assay.

The tissue leachate was obtained as follows: 1 ml of sterile PBS was added to the frozen tissues in storage tubes, and then the tissue was cut using sterile scissors in the biosafety cabinet. The tubes were centrifuged at 500 × *g* for 10 min. Then, 200 μl of the supernatant was used to isolate the virus. The virus separation from the tissue leachate was similar to serum virus separation procedure.

### Determination of the virus titre of organs

One millilitre of sterile PBS was added to the frozen tissues in storage tubes, and then the tissue samples were cut using sterile scissors in the biosafety cabinet. The tubes were then centrifuged at 500 × *g* for 10 min. Then, 200 μl of the supernatant was added with 800 μl Trizol to extract the RNA. Quantitative real-time PCR (qPCR) was then used to calculate the amount of the virus. qPCR was carried out using an H7N9 nucleic acid quantitative detection kit (Cat#Z-RR-0309-02; Zhijiang biological technology Co., Ltd. (Shanghai, China). Nineteen microlitres of H7N9 nucleic acid PCR detection reaction mixture and 1 μl of quantitative PCR enzyme were mixed by vortexing and then centrifuged at 500 × *g* for several seconds. This mixture was added to the PCR reaction tube, together with 5 μl of the RNA sample, for a total reaction volume of 25 μl. The tube was covered, centrifuged briefly, and subjected to the following PCR conditions: 40 cycles of 45 °C for 10 min, 95 °C for 15 s, and 60 °C for 60 s. The relative quantity of H7N9 virus was determined by the cycle threshold (Ct) value.

### Statistical analysis

GraphPad Prism 5 (GraphPad Software, Inc., La Jolla, CA, USA) was used for the statistical analyses of the survival rate and weight data. Statistical analyses of the data for weight was conducted using Mann–Whitney test. P-values < 0.05 were considered statistically significant.

## Results

### The change in weight and the survival status of the H7N9-infected mice

**Fig. 1:**
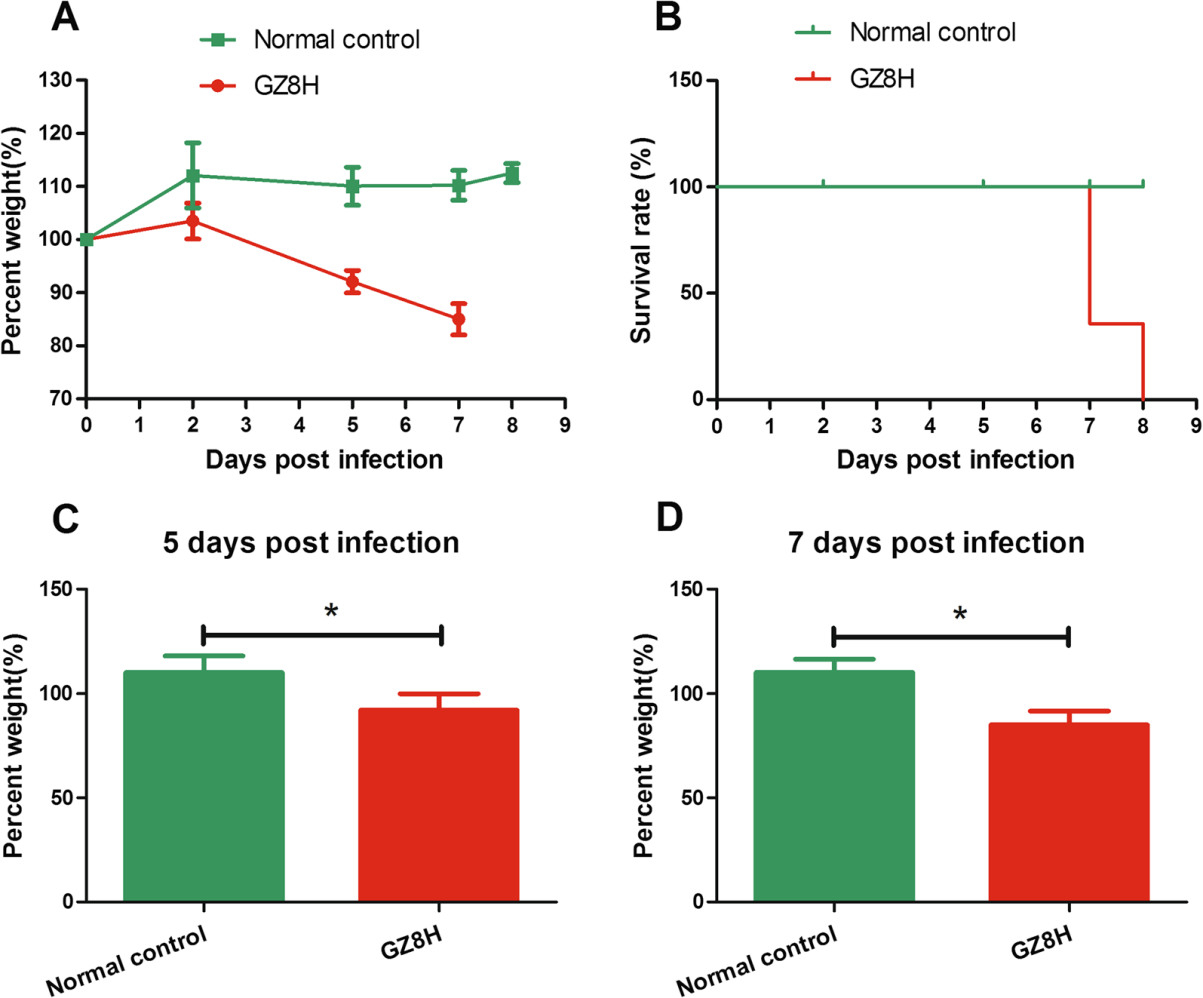
Changes in the weights of the experimental mice and their survival curve. **A** The weight change of the experimental mice. **B** The survival curve of the experimental mice. **C** The comparison of weights between the normal group and virus infection group 5 days post infection. **D** The comparison of weights between the normal group and virus infection group 7 days post infection. GZ8H: A/Guangdong/GZ8H002/2017(H7N9); *P-values < 0.05 were considered statistically significant

After H7N9 challenge, the mice lost weight, displayed signs of illness, and eventually died. Up to 2 dpi, the H7N9-infected mice showed mild illness, with a minor decrease in appetite and activity. The weight of the mice increased slightly over the first two days (Fig. 1A). However, subsequently, the weight of the mice decreased sharply (Fig. 1A, C, D), reaching a weight loss of nearly 20% (Fig. 1A). After 2 dpi, The health of the mice deteriorated gradually. The mice showed signs of a relatively acute clinical disease, manifested by poor appetite, ruffled fur, and inactivity. By 7 dpi, the death rate of the mice was more than 64% (Fig. 1B). The remaining live mice had signs of severe respiratory disease, such as respiratory distress and an increasing lack of appetite. By 8 dpi, all the mice had died (Fig. 1B). Throughout the observation period, the mice in the uninfected control group remained healthy, with no obvious loss of weight or illness.

### Histopathology of organs

**Fig. 2:**
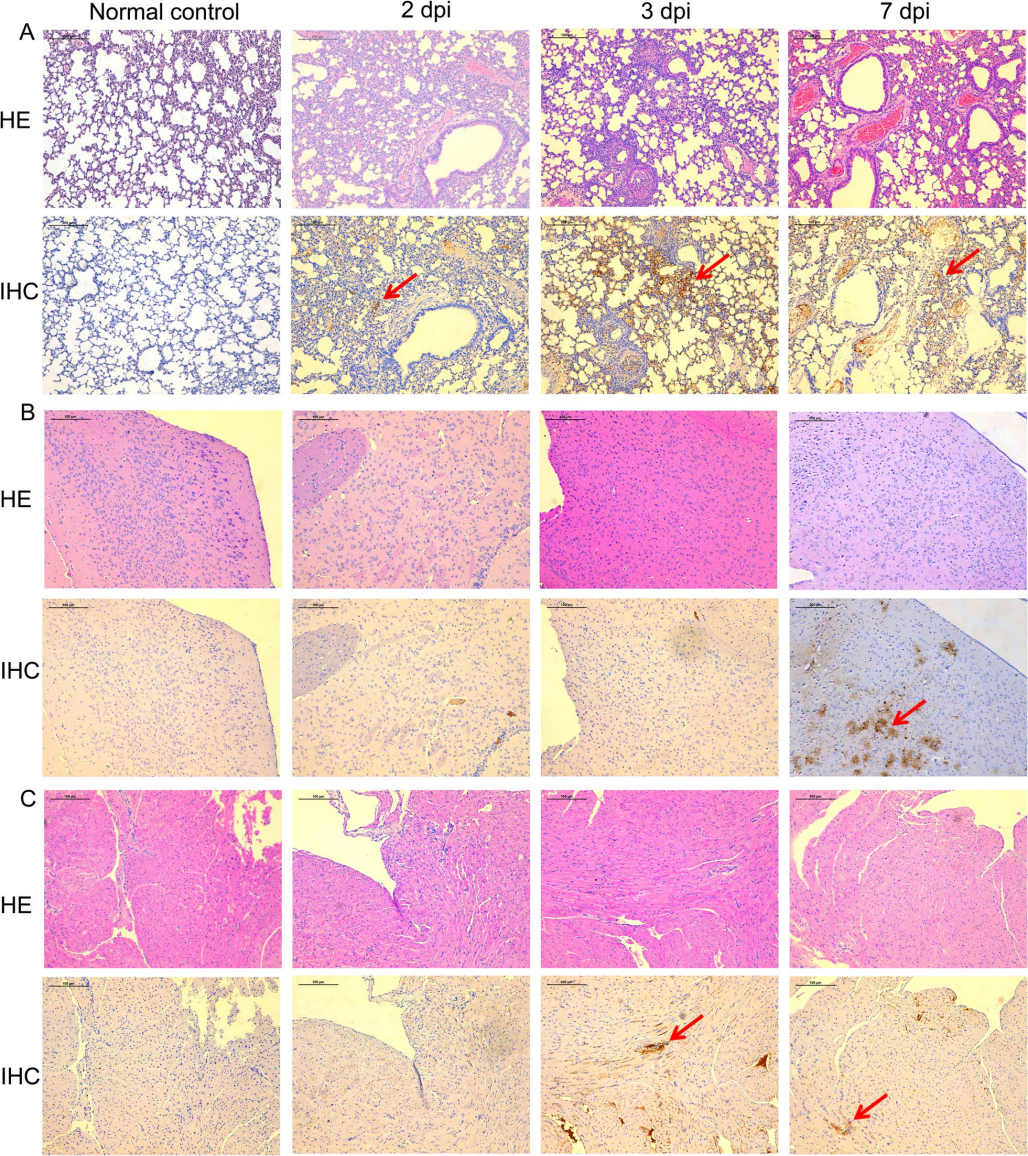
Histopathology of lung, brain, and heart tissue. **A** Immunological and HE staining of mouse lungs at 2, 3, and 7 days post infection. **B** Immunological and HE staining of mouse brains at 2, 3, and 7 days post infection. **C** Immunological and HE staining of mouse hearts at 2, 3, and 7 days post infection. The viral antigen is shown using a red arrow. HE: Haematoxylin and eosin; IHC: Immunohistochemistry; dpi: days post infection

HE staining was used to evaluate the histopathology of lung tissue. The number and appearance of the lesions varied at different dpi. At 2 dpi, the mice showed a lesion with inflammatory cell infiltration, exudation, and intra-alveolar haemorrhage. At 3 dpi, the mouse lungs showed pathological changes in the exudate and severe multifocal interstitial inflammatory hyperaemia. At 7 dpi, the lung lesions were larger compared with those at 2 and 3 dpi, and many patchy lesions had fused. At this time point, the intra-alveolar haemorrhage area was enlarged (Fig. 2A). As the dpi increased, varying degrees of lung injury were revealed using immunohistochemical staining. Lung tissue, particularly the bronchiolar epithelium, showed the presence of viral antigens. The mice at 3 and 7 dpi suffered severe injury, with many viral antigens being detected (Fig. 2A).

The brain tissue samples were also subjected to HE staining. As the dpi increased, the H7N9-infected showed more severe pathological changes. Brain IHC staining revealed abundant H7N9 antigens at 7 dpi (Fig. 2B). These results suggested that the brain was infected by the virus.

HE staining was also used to examine the heart histopathology. The number and appearance of the lesions varied according to the dpi. At 3 and 7 dpi, IHC staining revealed large amounts of H7N9 antigens in the heart (Fig. 2C). These results suggested that from 3 dpi, the heart became infected with the virus.

**Fig. 3:**
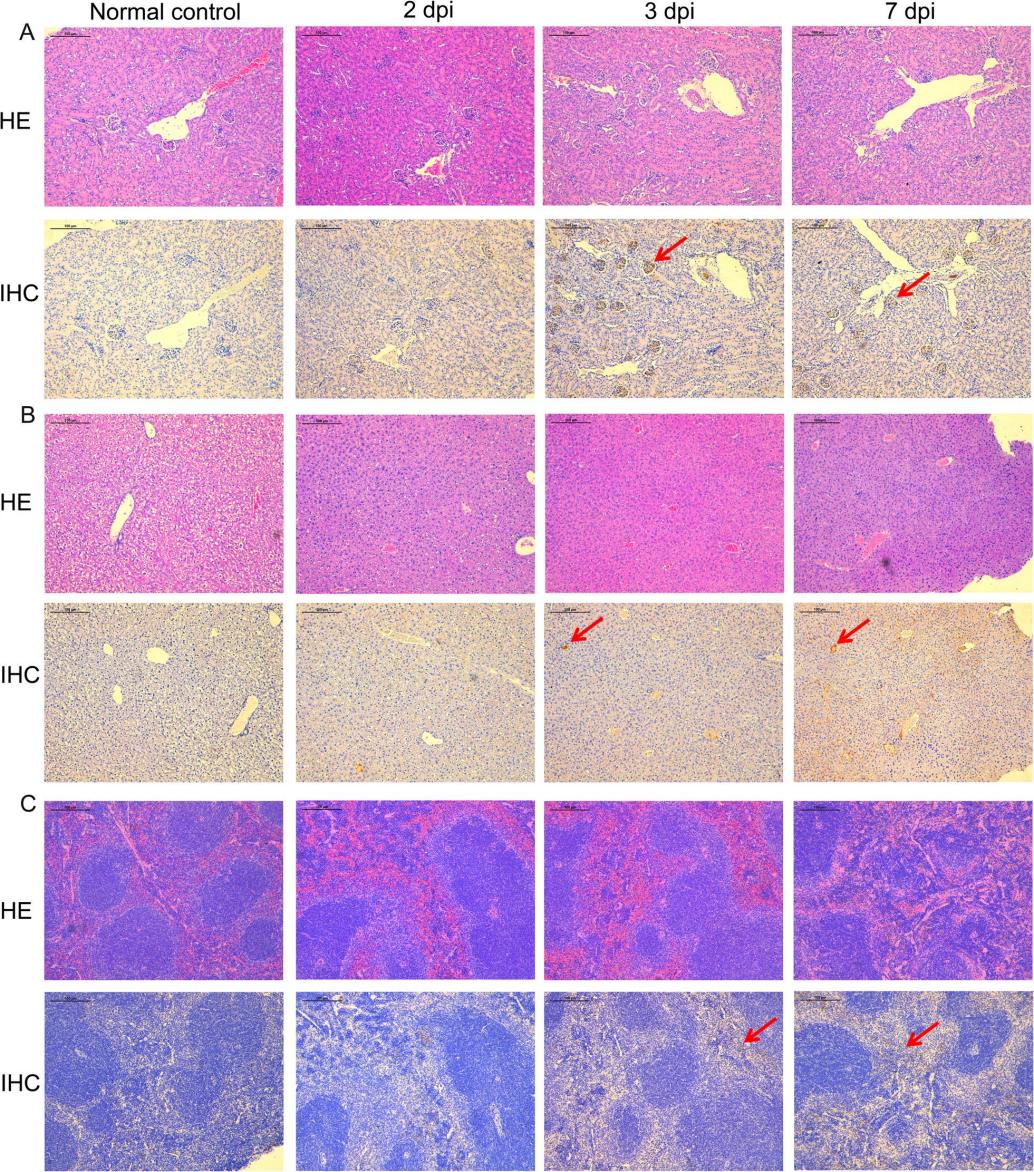
Histopathology of kidney, liver, and spleen tissue. **A** Immunological and HE staining of mouse kidneys at 2, 3, and 7 days post infection. **B** Immunological and HE staining of mouse livers at 2, 3, and 7 days post infection. **C** Immunological and HE staining of mouse spleens at 2, 3, and 7 days post infection. The viral antigen is shown using a red arrow. HE: Haematoxylin and eosin; IHC: Immunohistochemistry; dpi: days post infection

HE staining of kidney tissue revealed that H7N9 infection caused more severe pathological changes over time. At 3 and 7 dpi, IHC staining showed large quantities of H7N9 antigens in the kidneys (Fig. 3A). These results suggested that from 3 dpi, the kidneys became infected with the virus.

HE staining liver tissue was also revealed that the number and appearance of the lesions varied according to the dpi. At 3 and 7 dpi, IHC staining detected H7N9 viral antigens in the liver (Fig. 3B). These results suggested that from 3 dpi, the liver became infected with the virus.

HE staining of spleen tissue revealed that H7N9 infection caused more severe pathological changes over time. At 3 and 7 dpi, IHC staining detected H7N9 viral antigens in the spleen (Fig. 3C). These results suggested that from 3 dpi, the spleen became infected with the virus.

### Isolation of the virus from mouse serum and organs

Live H7N9 virus could be isolated from the serum of infected mice persistently until death (Table 1). At 2 dpi, H7N9 virus was isolated from the lung one of three mice. Virus could also be isolated from mice at 3 and 7 dpi. However, 2 and 3 dpi, no H7N9 virus could be isolated from brain samples. By contrast, at 7 dpi, H7N9 virus was isolated from all mouse brains. At 2 dpi, no H7N9 virus was isolated from heart samples, whereas at 3 dpi, virus could be isolated from the heart of one of three mice. At 7 dpi, the virus was isolated from three of six mouse hearts. At 2 dpi, no H7N9 virus could be isolated from the kidney samples. At 3 and 7 dpi, the virus was isolated from the kidney of one of three mice and two of six mice, respectively. The H7N9 virus was not isolated from the liver at 2 dpi. At 3 dpi and 7 dpi, virus was isolated from the liver of one of three and one of six mice respectively. At 2 dpi, no H7N9 virus could be isolated from the spleen samples. At 3 dpi and 7 dpi, virus was isolated from the spleen of one of three mice and two of six mice, respectively.

**Table 1:** The H7N9 virus isolated from serum and different tissues of mice

Tissue	Number of animals from which the virus was isolated		
	2 dpi (n = 3)	3 dpi (n = 3)	7 dpi (n = 6)
Lung	1	3	6
Brain	0	0	6
Heart	0	1	3
Kidney	0	1	2
Liver	0	1	1
Spleen	0	1	2
Serum	1	3	6

Dpi, days post infection

### The amount of virus in mouse organs

**Fig. 4:**
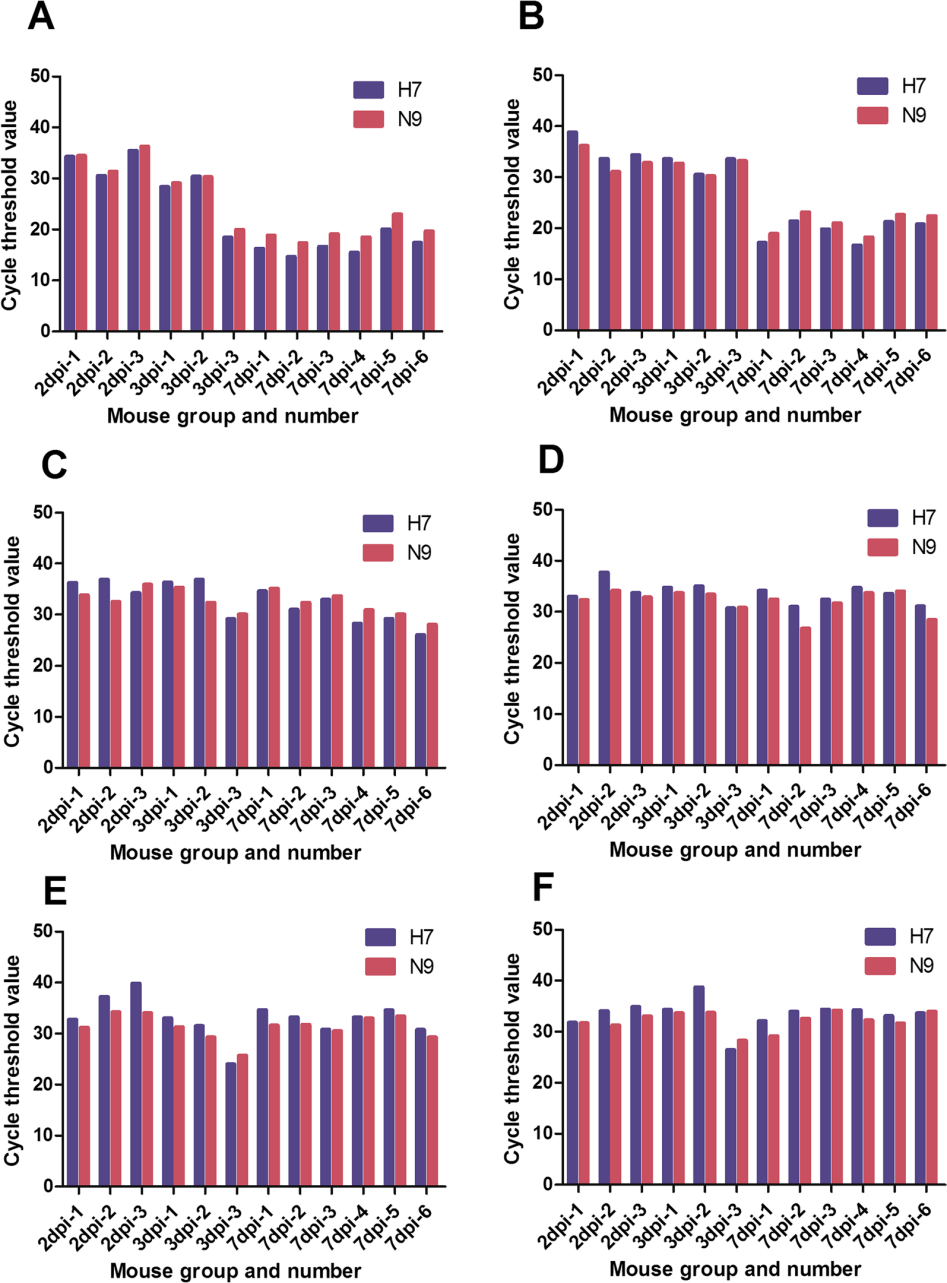
Reverse transcription-polymerase chain reaction results in different tissues of each group of mice. **A** Reverse transcription-polymerase chain reaction results in lungs of each group of mice. **B** Reverse transcription-polymerase chain reaction results in brains of each group of mice. **C** Reverse transcription-polymerase chain reaction results in hearts of each group of mice. **D** Reverse transcription-polymerase chain reaction results in kidneys of each group of mice. **E** Reverse transcription-polymerase chain reaction results in livers of each group of mice. **F** Reverse transcription-polymerase chain reaction results in spleens of each group of mice. dpi: days post infection; The H7 and N9 gene levels are shown as the cycle threshold (Ct) value; A Ct value > 38 was considered negative

Quantitative PCR was used to evaluate the amount of virus in mouse organs. The RNA level was represented by the Ct value. A Ct value > 38 was considered negative. The lower the Ct value, the higher the virus content. The RNA level was relatively low in mouse lungs at 2 dpi, but increased sharply at 3 dpi and again at 7 dpi (Fig. 4). The RNA level was relatively low in the brains of the mice at 2 and 3 dpi, but increased to a high level at 7 dpi. The RNA level was relatively low in the hearts of mice at 2 dpi. However, the virus content in the heart was relatively high in one of three mice at 3 dpi and in three of six mice at 7 dpi. The RNA level was relatively low in the kidneys of the mice at 2 dpi. However, the virus content in the kidney was relatively high in one of the mice at 3 dpi and in two of six mice at 7 dpi. The RNA level was relatively low in the livers of the mice at 2 dpi. However, the virus content in the liver was relatively high in one of three mice at 3 dpi and in one 1 of six mice at 7 dpi. The RNA level was relatively low in the spleens of mice at 2 dpi. However, the virus content in the spleen was relatively high in one of three mice at 3 dpi and in two of six mice at 7 dpi.

## Discussion

We have confirmed that viremia was associated with the high mortality caused by highly pathogenic H7N9. Lung infection is the main manifestation, and the virus eventually causes multiple organ failure. Multiple organ failure might have many causes, including a systemic inflammatory response resulting from pulmonary infection [35], but might also be caused by late multiple organ infection. In the present study, we found that in mice infected with the A/Guangdong/GZ8H002/2017(H7N9) virus. Their weight decreased sharply from 3 dpi. Our results suggested that in the early phase of infection (up to 2 dpi), lung lesions were few and the virus titre was low. During this early period, the other organs were not infected. The first two days is a very important time window for early diagnosis and treatment. In a previous study of in patients with H7N9 viral infection, the early use (in the first 48 h of illness) of a neuraminidase inhibitor decreased the duration of viral shedding and improved survival [36]. In addition, as the virus has changed, the time of antiviral use has been extended to fully control the infection [36]. Thus, in highly pathogenic H7N9 virus infection, antiviral treatment should be started as early as possible. We observed that on the third day post infection, the disease burden increased sharply, enhancing the risk of multiple organ infection, which would require a longer period of antiviral therapy and might result in multiple organ injury.

In our previous study, the H7N9 virus was not lethal to mice [34]. In other previous studies, the highly pathogenic H7N9 viruses isolated from the avian species could replicate in the airway of mice but were no lethal to mice [5]. However, with the evolution of H7N9 highly pathogenic viruses, some of the viruses became highly virulent in mice. Recent studies have shown that after circulation in poultry for a few weeks, some viruses acquired the ability to kill mice [13]. Once the virus obtained the PB2 701 N or PB2 627 K mutation, its virulence in mice could increase over 10,000-fold [5, 13, 14]. In another study, the authors using the highly pathogenic H7N9 viruses isolated from the avian species between 2018 and 2019 to infect the mice, some mice were detected with brain and spleen infection, but the authors have not explore the infection in other major organs like hearts, kidneys and livers [14]. However, in our present study, all the mice infected with highly pathogenic H7N9 were dead by the 8th day post infection with multiple organ injuries.

K526R in PB2 gene can increase pathogenicity to mice in the absence of E627K and D701N mutations [37]. M535L in PB2 gene has been reported to increase viral polymerase activity [38]. Having both K526R and M535L mutations in PB2 gene might be responsible for the increased viral virulence of A/Guangdong/GZ8H002/2017(H7N9) virus, which required further verification by reverse genetic techniques. The highly pathogenic H7N9 virus induced a more enhanced immune response than the low pathogenic H7N9 virus. Pathogenicity of high pathogenic H7N9 was increased by inducing a stronger cytokine storm [39]. The cause of death in mice is considered to be a result of increased replication capacity of highly pathogenic H7N9 virus, which is observed as rapid viral replication in lungs of mice on 3–4 dpi, the lung blood barrier breaking, viremia and other organs infection, later multi-organ infection and failure [39]. It simultaneously induced a cytokine storm, resulting in multi-organ function impairment [39]. Direct damage caused by high pathogenic H7N9 virus and immune damage together contribute to death in mice.

Heart, kidney, and liver injuries are common in influenza infection [22, 40, 41]. Moreover, influenza A (H1N1) has been detected in patients’ pericardial and myocardial tissues [42, 43]. These studies suggest that infection-associated cardiac injury is caused by direct viral invasion. Heart changes have also been reported during H7N9 infection. For example, in 203 out of 321 patients, evidence of cardiac injury was observed [44]. Cardiac injury was associated with severe infections, acute respiratory distress syndrome, and the associated settings of the mechanical ventilator and was linked higher mortality during hospitalization [44]. In our study, heart infection was confirmed in mice. Heart infection might also be the cause of high rates of pathogenic H7N9 mortality. Therefore, we should closely monitor myocardial function in patients infected with highly pathogenic H7N9.

In our study, during the late stage of infection, all the mice showed brain infection. Influenza-associated encephalopathy might be another important cause of death in patients with influenza [45]. Seasonal and pandemic H1N1 infections in 2009 were found to cause encephalopathy in patients [46, 47]. In a previous study, the highly pathogenic H7N9 virus exhibited enhanced virulence and extended viral tropism in mice compared with low pathogenic H7N9 viruses [48]. Meanwhile, the highly pathogenic H7N9 virus has been proven to replicate in brain tissues in animal models [48]. H5N1 virulence in mice correlates with that in humans; therefore, we could speculate that highly pathogenic H7N9 strains that caused fatalities in mice would also show increased lethality in humans [12, 13]. When the patients develops multiple organ injuries and brain infection, they should receive urgent intensive care treatment. In the intensive care unit, we should maintain organ function, strengthen antiviral treatment, and provide life support treatment. Such comprehensive treatment can save patients’ lives [4, 49]. At the same time, we should pay attention to a patient’s intracranial pressure and note any changes to their mental capacity [50].

## Conclusions

In summary, influenza virus mutations increase infection rates in humans and may also lead to pathogenic enhancement. We should closely monitor the H7N9 virus because it might gain greater multi-organ infection capacity through mutations, leading to higher fatality rates. Acute diffuse lung injury is the initial stage of pathogenesis in highly pathogenic H7N9 virus infection. In addition to lung infection and viremia, the highly pathogenic H7N9 virus can cause multiple organ infection and injury, which should receive more research attention. Therefore, early diagnosis, early use of drugs or neutralization antibodies to control the virus, and the prevention and control of multiple organ infection are very important.

## Abbreviations

PB2: Polymerase basic protein 2
SPF: Specific-pathogen-free
MDCK: Madin-Darby canine kidney
DMEM: Dulbecco's modified Eagle's medium
FBS: Foetal bovine serum
HA: Haemagglutinin
PBS: Phosphate-buffered saline
TCID_50_: Median tissue culture infectious dose
dpi: Days post infection
HE: Haematoxylin eosin
IHC: Immunohistochemistry
qPCR: Quantitative real-time PCR
Ct: Cycle threshold

## Declarations

### Availability of data and materials

All data generated or analysed during this study are included in this published article.
